# 

**Published:** 1918-04-20

**Authors:** 


### Manager's Notices.

**Rates of Subscription** (payable in advance).

UNITED KINGDOM.

| | | |
|---|---|---|
| Three Months | (including Postage) ... | 2s. 0d. |
| Six Months | do. ... | 4s. 0d. |
| Twelve Months | do. ... | 6s. 6d. |

FOREIGN AND COLONIAL.

| | | |
|---|---|---|
| Three Months | (including Postage) ... | 2s. 6d. |
| Six Months | do. ... | 5s. 0d. |
| Twelve Months | do. ... | 8s. 8d. |

**All letters on business matters must be addressed to The Manager, "The Hospital," 28 and 29 Southampton Street, London, W.C. 2.**

**Special Rates** will be quoted to those institutions that agree to send all their public notices to The Hospital, so as to make it a file of such announcements for ready reference.

### THE COLLEGE OF NURSING

(Limited by Guarantee).

### List No. 6 of Training Schools of Registered Nurses.

At the last Council meeting of the College of Nursing applicants for membership were passed holding certificates of the following training-schools :—

Great Northern Central Hospital, N.; Guy's Hospital, S.E.; Hampstead General Hospital, N.W.; King's College Hospital, S.E.; London Hospital, E.; London Temperance Hospital, N.W.; Metropolitan Hospital, N.E.; Miller General Hospital, Greenwich; St. Bartholomew's Hospital, E.C.; St. George's Hospital, S.W.; St. Thomas's Hospital, S.E.; Queen Mary's Hospital, Stratford; Westminster Hospital, S.W.; Bermondsey Poor-Law Infirmary, S.E.; Chelsea Poor-Law Infirmary; Hackney Union Infirmary; Lambeth Poor-Law Infirmary, S.E.; Mile End Poor-Law Infirmary; St. George's Poor-Law Infirmary; St. George-in-the-East Poor-Law Infirmary; St. Leonard's Poor-Law Infirmary, Shoreditch; St. Mary's Poor-Law Infirmary, N.; Southwark Union Infirmary, S.E.; Whitechapel Poor-Law Infirmary, E.; Beckett Hospital, Barnsley; County Hospital, Bedford; Borough Hospital, Birkenhead; General Hospital, Birmingham; Royal Infirmary, Blackburn; The Infirmary, Bolton; Royal Infirmary, Bristol; General Infirmary, Burton-on-Trent; Addenbrooke's Hospital, Cambridge; Kent and Canterbury Hospital, Canterbury; Royal Infirmary, Chester; Essex and Colchester General Hospital, Colchester; Coventry and Warwickshire Hospital, Coventry; County Hospital, Durham; Royal Infirmary, Gloucester; Royal Infirmary, Halifax; Hertford County Hospital, Herts; Royal Infirmary, Hull; General Infirmary, Leeds; Royal Infirmary, Leicester; Royal Infirmary, Liverpool; Royal Southern Hospital, Liverpool; Stanley Hospital, Liverpool; West Kent General Hospital, Maidstone; Royal Infirmary, Manchester; Royal Hospital, Salford; Royal Victoria Infirmary, Newcastle-on-Tyne; General Hospital, Nottingham; General Hospital, Northampton; Norfolk and Norwich Hospital, Norwich; Royal Infirmary, Oldham; Radcliffe Infirmary, Oxford; South Devon and East Cornwall Hospital, Plymouth; Royal Berks Hospital, Reading; St. Helen's Hospital, Peasley Cross; The Infirmary, Salisbury; Royal Infirmary, Sheffield; Royal Salop Infirmary, Shrewsbury; Staffordshire General Infirmary, Stafford; North Staffs Infirmary, Stoke-on-Trent; General Hospital, Swansea; Royal Cornwall Infirmary, Truro; Princess Christian Hospital, Weymouth; Wolverhampton and Staffs General Hospital, Wolverhampton; County Hospital, York; Stapleton Poor-Law Infirmary, Bristol; Union Infirmary, Cardiff; Medway Union Infirmary, Chatham; North Evington Poor-Law Infirmary, Leicester; Kingston Poor-Law Infirmary, Kingston-upon-Thames; Brownlow Hill Poor-Law Infirmary, Liverpool; West Derby Union, Alder Hay Hospital; West Derby Union, Highfield Infirmary; West Derby Union, Mill Road Infirmary; West Derby Union, Walton Infirmary; Crumpsall Poor-Law Infirmary, Manchester; Withington Poor-Law Hospitals, West Didsbury; Union Infirmary, Salford; Bagthorpe Poor-Law Infirmary, Nottingham; Union Infirmary, Prescot; Poor-Law Infirmary, Portsmouth; Rochdale Union Hospitals; Firvale Union Hospital, Sheffield; Union Hospital, Stoke-upon-Trent; Steyning Poor-Law Infirmary, Shoreham; Harton Union Hospitals, South Shields; Union Infirmary, Swansea; Union Infirmary, Tonbridge; West Bromwich and Union Infirmary; Wolstanton and Burslem Union Infirmary; St. Joseph's Hospital, Victoria, British Columbia; Seamen's Hospital, Greenwich, and Hospital for Women, Soho, W.; Brompton Hospital, S.W., and Royal Infirmary, Leicester; Brompton Hospital, S.W., and General Hospital, Birmingham.

### Matrons' and Sisters' Appointments.

Children's Hospital, Lower Sydenham, S.E.—Miss Janet Raymond has been appointed theatre and surgical sister. She was trained at Queen's Hospital for Children, Hackney Road, N.E., has been charge nurse at Hanwell Schools, and sister at the Children's Infirmary, West Norwood.

County Hospital, York.—Miss Mary Thwaites has been appointed ward sister. She was trained at Blackburn and East Lancashire Royal Infirmary, and has since been sister at the Victoria Hospital, Keighley, at the Victoria Hospital, Romford, at Gravesend General Hospital, and at Chaseside Military Hospital, St. Anne's-on-Sea.

Earlsmead Home of Recovery, Hornsey Lane (Great Northern Central Hospital Branch).—Miss Helen K. Hawkins has been appointed matron. She was trained at the Great Northern Central Hospital, where she was later sister, and has been matron of Winifred House Children's Home Hospital, Tollington Park.

Eye and Ear Infirmary, Liverpool.—Miss Phyllis M. Lea has been appointed sister. She was trained at the North Derbyshire Hospital, Chesterfield, and has since been sister at Surbiton General Hospital and at Macclesfield General Infirmary. She has also done private nursing.

Great Northern Central Hospital, Earlsmead Home of Recovery.—Miss Helen K. Hawkins has been appointed matron. She was trained at the Great Northern Hospital, where she was later sister. She has since been matron of the Winifred House Children's Hospital, Tollington Park.

Royal City of Dublin Hospital.—Miss Eileen Rohde has been appointed matron. She was trained at the Royal City of Dublin Hospital, where she later served on the private staff, was sister of the fever wing, and temporarily acted as home sister. In 1909 she entered the service of the Egyptian Government at the Kasr-el-Aini Hospital, Cairo, where she acted as night superintendent. She was appointed matron of the military hospital of Trimulgherry, Secunderabad, India, after acting for a short time as sister at the military hospital at Peshawar. Miss Rohde, who obtained First Class Honours at her training-school, holds the certificates of the Central Midwives Board and the Incorporated Society of Trained Masseuses.

Royal Cornwall Sailors' Hospital, Falmouth.—Miss Louise Jackson has been appointed matron. She was trained at the General Hospital, Northampton, and at the Fever Hospital, Farnworth, Bolton; has been night sister and deputy matron at the Royal Hamadryad Seamen's Hospital, Cardiff, ward sister at the Welsh Metropolitan War Hospital, Whitchurch, near Cardiff, and has also done private nursing.

St. Chad's Hospital, Birmingham.—Miss E. Truslove has been appointed matron. She was trained at Queen's Hospital, Birmingham, has been staff and district nurse at Hednesford Accident Hospital, sister, and later matron of Keighley and Bingley Isolation Hospital, and matron of Smallwood General Hospital, Redditch. She has also done nursing in France.

4th Auxiliary Hospital, Moseley, Birmingham.—Miss F. A. Gothard has been appointed night sister. She was trained at the General Hospital, Birmingham.

**NOTE.—Particulars of Appointments for insertion in this column will be welcomed.**

### Gifts and Bequests.

Mr. J. T. Fair, of Lytham, stockbroker, left £100 to Lytham Cottage Hospital and £50 to Lytham Parish Nursing Fund.

Putney Hospital has been left £200 Barnet District Gas and Water Company's Debenture Stock under the will of the late Mr. Alfred Lass, of Putney.

Under the will of Mr. Joseph Turner Bailey, of Cambridge, formerly a Bath-chairman, about £10,000 is bequeathed to Addenbrooke's Hospital, Cambridge.

Four members of the committee of King Edward Memorial Cottage Hospital, Hendon, have conditionally promised £400 towards the provision of further beds.

At a recent meeting of the Board of Governors of the Manchester Royal Infirmary it was reported that a legacy of £500 had been received from the executors of the late Mr. Arthur Lavington Payne.

Mr. John Reid has given the Princess Louise Hospital for Limbless Soldiers at Erskine a wood of fifty acres and an area of forty-five acres of agricultural land adjoining. The hospital grounds now extend to 500 acres.

A bed in memory of Mr. C. H. Barnwell has been established in Weybridge Cottage Hospital, towards which Messrs. Vickers, Ltd., have given £500. Mr. Barnwell was employed by Messrs. Vickers, and was killed whilst testing a new machine in a Kentish aerodrome.

Mr. John Raynar, of Scarborough, has left £500 each to the Leeds General Infirmary, the Scarborough Hospital and Infirmary, and the National Children's Home and Orphanage, London. Dr. Barnardo's Homes and the Scarborough District Nursing Association have been left £250 each.

In memory of his son, Captain A. F. Chance, Mr. F. W. Chance, ex-M.P. for Carlisle, has given the Carlisle Education Committee a sum of £4,000 for the purpose of establishing a laboratory and lecture-room for the teaching of chemistry and physics, and more especially for the promotion of research work.

Under the will of the late Mr. William Brechin Faulds, a solicitor, of Glasgow, four scholarships of £200 a year have been founded in arts, medicine, divinity, and law in the University of Glasgow. Each is tenable for three years, and it is probable that the first election will be postponed until candidates now on military service shall have returned.

After the death of his widow, Mr. Nathaniel Hone, an Irish artist who was associated with Corot, Millet, and others of the Barbizon school, has left £200 each to Meath Hospital, Sir Patrick Dun's Hospital, the Victoria Eye and Ear Hospital, Dublin, and the National Orthopædic Hospital, Dublin, and £100 to Harcourt Street Hospital for Sick Children, Dublin.

Two welcome donations of £1,000 each have been received by the Royal Gwent Hospital, Newport. One is from Mr. and Mrs. Thomas Spittle, of Cambrian House, Maindee, to endow a bed in memory of their elder son, the late Captain Thomas Stanley Spittle, who was killed in action during October 1917. A bed in memory of Captain Spittle has also been endowed in the Prince of Wales' Hospital, Cardiff, by a gift of 1,000 guineas from his uncle and aunt, Sir Thomas and Lady Watson. The second £1,000 cheque received by Mr. J. K. Millward, secretary of the Royal Gwent Hospital, is from Alderman Frederick Phillips, J.P., Provincial Grand Master of the Monmouthshire Province of Freemasons. This bed is to be known as the Monmouthshire Freemasons'.

Sir John Wolfe Barry left £1,000 to Westminster Hospital and a further £1,000 after the death of Lady Barry.

A Bristol Lady has sent to the Royal Hospital and Home for Incurables, Putney Heath, £500 National War Bonds for establishing a pension for an incurable invalid.

Mrs. Margaret Kilner has bequeathed £1,000 to the Huddersfield Orphan Homes. The residue of the estate is to be divided between the Victoria Sick Poor Nurses' Association, the Charity Organisation Society, and other charities.

Mr. C. E. S. Cockburn, of Lancaster Gate, W., has left shares in the Staveley and District Public House Trust to Chesterfield Hospital, the Derby Royal Infirmary, the Devonshire Hospital, Buxton, the London Hospital, and the National Hospital for the Paralysed and Epileptic. We are informed that the shares are of little or no value.

Mrs. Susannah Bowen, of Beccles, Suffolk, left £300 to the Fishermen's Hospital, Great Yarmouth. After certain family bequests the testatrix left one third of the residue of her estate to such charitable institutions and societies as the trustees of the will shall determine.

---

Over £1,000, or £270 more than last year, has been realised by the Hospital Day collections in the various parishes of the Neath Union.

### Personalia.

Dr. Robert A. Lyster, M.D., Ch.B., B.Sc., D.P.H., Lecturer in Public Health and Forensic Medicine at St. Bartholomew's Hospital, E.C. (London University), and County Medical Officer for Hampshire, has been elected to the editorship of *Public Health*, the official journal of the Society of Medical Officers of Health, in place of Lieut.-Colonel Sir Shirley F. Murphy, R.A.M.C., F.R.C.S., who resigned recently.

The Hull Royal Infirmary sustained a heavy loss during last month by the death of two valued members of its staff, in the person of Mr. R. H. B. Nicholson, consulting surgeon, who had been connected with the institution for forty-two years, and Mr. J. C. Storey, consulting dental surgeon. Sympathetic reference was made to their passing away by Sir James Reckitt, in presiding at the infirmary's annual meeting on March 28.

### The Book Market.

LIST OF NEW BOOKS RECEIVED FROM THE FOLLOWING PUBLISHERS:—

Society for Promoting Christian Knowledge: "The Baby." By Sophia Seekings, M.D., etc. 9d. net.

John Bale, Sons and Danielsson, Ltd.: "The Panel Doctor: His Duties and Perplexities." By T. M. Tibbetts, M.D. Lond. 2s. 6d. net. "Infant Welfare." A Model Infant Welfare Centre. 24 Perforated Picture Postcards. 1s. net.

---

Messrs. J. and A. Churchill will shortly publish a translation by Dr. Mina L. Dobbie from the Swedish of the well-known book on massage and medical gymnastics by Dr. Emil A. G. Kleen. The section on medical gymnastics is by Dr. J. Arvedson. Dr. Dobbie is medical officer to the Chelsea Physical Training College. Numerous illustrations elucidate the letterpress.

